# Optimised and Rapid Pre-clinical Screening in the SOD1^G93A^ Transgenic Mouse Model of Amyotrophic Lateral Sclerosis (ALS)

**DOI:** 10.1371/journal.pone.0023244

**Published:** 2011-08-18

**Authors:** Richard J. Mead, Ellen J. Bennett, Aneurin J. Kennerley, Paul Sharp, Claire Sunyach, Paul Kasher, Jason Berwick, Brigitte Pettmann, Guiseppe Battaglia, Mimoun Azzouz, Andrew Grierson, Pamela J. Shaw

**Affiliations:** 1 Sheffield Institute for Translational Neuroscience, Department of Neuroscience, School of Medicine and Biomedical Sciences, University of Sheffield, Sheffield, United Kingdom; 2 Department of Psychology, Faculty of Science, University of Sheffield, Sheffield, United Kingdom; 3 Inserm-Avenir Team, Mediterranean Institute of Neurobiology, Inmed, Marseille, France; 4 Faculté des Sciences, Aix Marseille Université, Marseille, France; 5 Department of Biomedical Sciences, University of Sheffield, Sheffield, United Kingdom; Biological Research Center of the Hungarian Academy of Sciences, Hungary

## Abstract

The human SOD1^G93A^ transgenic mouse has been used extensively since its development in 1994 as a model for amyotrophic lateral sclerosis (ALS). In that time, a great many insights into the toxicity of mutant SOD1 have been gained using this and other mutant SOD transgenic mouse models. They all demonstrate a selective toxicity towards motor neurons and in some cases features of the pathology seen in the human disease. These models have two major drawbacks. Firstly the generation of robust preclinical data in these models has been highlighted as an area for concern. Secondly, the amount of time required for a single preclinical experiment in these models (3–4 months) is a hurdle to the development of new therapies. We have developed an inbred C57BL/6 mouse line from the original mixed background (SJLxC57BL/6) SOD1^G93A^ transgenic line and show here that the disease course is remarkably consistent and much less prone to background noise, enabling reduced numbers of mice for testing of therapeutics. Secondly we have identified very early readouts showing a large decline in motor function compared to normal mice. This loss of motor function has allowed us to develop an early, sensitive and rapid screening protocol for the initial phases of denervation of muscle fibers, observed in this model. We describe multiple, quantitative readouts of motor function that can be used to interrogate this early mechanism. Such an approach will increase throughput for reduced costs, whilst reducing the severity of the experimental procedures involved.

## Introduction

Since 1994, transgenic mice expressing high levels of mutated human SOD1 have been used as a model of amyotrophic lateral sclerosis [1]. These mice replicate the selective vulnerability of motor neurones seen in the human disease. Muscle fibres become denervated, axons degenerate and spinal motor neurones undergo cell death [2]. Despite sprouting and partial re-innervation of muscle fibres by slow motor neurones [1], [3], muscle volume decreases [4] with increasing paralysis to the point where humane intervention is required.

Many advances in our understanding of ALS have emerged from studies in mutant SOD1 transgenic mice. In this model, various pathologic changes and mechanisms have been elucidated including mitochondrial abnormalities [5], oxidative stress [6], [7] glutamatergic toxicity [8], [9], cytoskeletal dysfunction [1], [10], [11], [12], [13], defective axonal transport [13], [14], [15] and repeated denervation and reinnervation of neuromuscular junctions [2], [3], [16], [17].

Although studies in human ALS patients are critical, one weakness of such experiments is the requirement to use post-mortem CNS material collected at the end stage of disease. An obvious advantage of studies in mouse models is the availability of CNS material throughout the lifespan of the mice. Genetic manipulation enables testing of novel hypotheses, for example, targeted expression of mutant SOD and the use of chimaeric mice has shown that expression of mutant SOD1 in astrocytes and microglia is an important driving force in disease progression [18].

The other major application of these murine models is in preclinical testing of new therapies. The utility of these mouse models for this purpose has been called into question [19]. Many drugs and therapeutic approaches have been tested, with the majority of interventions reported showing relatively mild effects and translation of these therapeutic benefits to the human disease has so far been unsuccessful [20]. Much of the criticism has been leveled at the poor quality of the preclinical study design. The ALS therapy development institute (ALSTDI), a non-profit organisation established to rapidly screen potential therapies in the SOD1^G93A^ mouse model, have tested >70 compounds in 221 studies using over 18, 000 mice [21]. They have been unable to replicate any of the published positive effects of compounds in this model. Using their own data to identify possible sources of noise and bias, they concluded that the majority of published positive neuroprotective effects were in fact due to biological noise and poor study design [21].

The 142^nd^ European NeuroMuscular Centre Workshop on ‘Guidelines on Preclinical models of ALS’ adopted the ALSTDI recommendations for the conduct of pre-clinical studies which included the use of litter matched control and treatment groups, n numbers of 24 per group, gene copy number analysis for all mice on therapeutic trials and the censoring of data from littermates of mice which are lost from the study due to non-ALS related events [22]. These guidelines have been recently updated to include a checklist for reviewers and journal editors when assessing studies in this model [23].

The commonly used SOD1^G93A^ strain is maintained by crossing hemizygous male transgenic mice with female C57BL6×SJL/J hybrids. This leads to genetic variation in these mice and genetic variation is known to increase variability in biological readouts. The best way of avoiding this noise is to use well defined inbred mouse strains [24], [25]. We have pursued a strategy of using the SOD1^G93A^ transgenic mice on an inbred, C57BL/6 genetic background to minimise genetic variation and enhance the quality of data generated in this model. We have gathered data in multiple therapeutic trials using a newly developed behavioural assessment protocol and show here that our model is very consistent across studies and less variable than the published data for the models on a mixed genetic background, reducing biological noise and consequently the number of mice required to reach statistical significance in efficacy studies.

We have also detected early behavioural changes (reduced rotarod performance) prior to the previously reported onset of clinical signs, which correlate remarkably well with the described pattern of early dennervation and re-innervation described in this model [3]. This has led to the development of further quantitative measures of early disease progression (automated gait analysis and quantification of hind limb muscle volume using magnetic resonance imaging) and the design of a ‘rapid early’ screening protocol. This refinement has the combined benefit of being faster, less resource intensive and less distressing to the mice, satisfying the underlying principles of UK regulated animal research which is embodied in the principles of reduction, refinement and replacement (the 3Rs) first proposed by Russell and Burch [26]. In addition it enables precise evaluation of therapeutic benefit with respect to the early denervation seen in this model and also described in man [2]. We expect that our approach and the model that we have generated will be of value to the ALS research community, helping to accelerate development of therapeutic candidates for effective clinical translation and will inform approaches to pre-clinical testing in other neurological disease areas also.

## Results

We conducted six pharmacology studies in the C57BL/6 SOD1^G93A^ transgenic mouse model of ALS over a two year period, representing five small molecule drug studies and one gene therapy study. The details of each study are shown in table 1 and the vehicle control groups were used for the analysis of variability below. At the outset we applied principles of good experimental design based on publications such as ‘The Design of Animal Experiments’ [27]. We were also aware of the need to monitor transgene copy number in our studies, as reductions in copy number can occur within breeding colonies and significantly alter expression of the disease course. Indeed, transgene copy number correlates directly with survival [28]. We routinely collected data for transgene copy number in male breeders and in all mice on pharmacology studies initially. No significant variation was observed for mice on pharmacology studies, however, reduction in copy number was detected in the breeding colony on two occasions (∼1/year). These mice were identified readily by their phenotype at 90 days of age. Since these events were rare and easily identified by phenotype, we have subsequently only conducted copy number analysis where a reduced copy number is suspected from the phenotype.

**Table 1:** Summary of the control group data in the C57BL/6 SOD1^G93A^ transgenic mice.

Study reference number	Dose	Route and frequency	N	Age at first dose	Start date
1	Vehicle	Once daily gavage (10 ml/kg)	9	45	13/4/05
2	Vehicle	Intraperitoneal, 3/week (5 ml/kg)	13	45	13/05/05
3	Vehicle	Once daily gavage (10 ml/kg)	10	75	14/7/05
4	Vehicle	In water (continuously) and subcutaneous (3/week, 5 ml/kg)	12	45	23/02/06
5	Control virus	Multiple intramuscular injections (120 µ1 total, 5 µl/site), once at 21 days of age	10	21	10/10/06
6	Vehicle	Chow continuously	11	25	23/03/07

**Figure 1:**
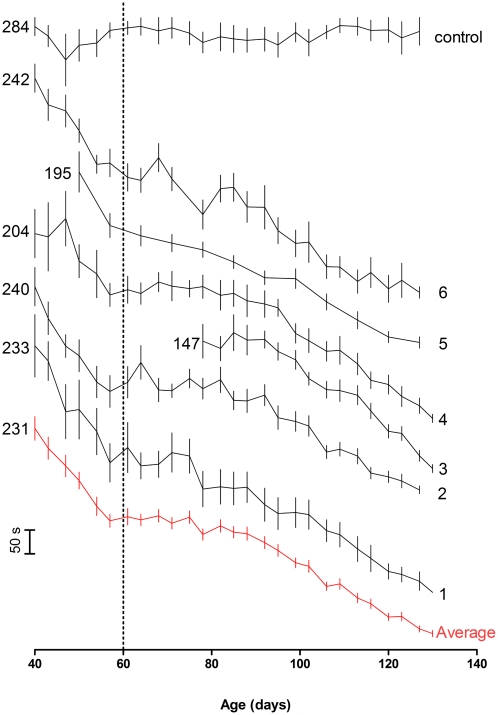
SOD1^G93A^ mice on a C57BL/6 background show a consistent, early decline in rotarod performance. Rotarod performance measured as time to fall in seconds (y axis, scale bar at left) +/− standard error of the mean (vertical bars) against age in days for six control groups from independent efficacy studies in the SOD1^G93A^ transgenic mouse model of ALS. Study details are given in table 1. The data are offset to allow comparison of the individual curves, the number at the left hand side of the curve is the average time in seconds for the first data point. Also shown are data from a group of control animals (non-transgenic littermates of mice in study number 6) and a plot of the average data for the six control groups (average). Curves are labelled at the right hand side with study number, ‘control’ and ‘average’. A dotted reference line is shown at 60 days, where the rate of decline in performance slows considerably for most studies.

A total of 6 therapeutic studies were performed in the C57BL/6 SOD1^G93A^ transgenic mouse model of ALS over approximately 2 years. Reference number refers to the data shown in table 2 and figure 1. Studies were conducted according to a standardised protocol as described in the materials and methods.

We included a number of readouts, including motor function (rotarod test), weight, disease onset (first signs of tremor and hind-limb splay defect) and survival (loss of righting reflex for more than 10 seconds or 30% weight loss for 72 h) as well as scoring the overall distress level in mice from 125 days of age onwards. These readouts were kept as standardised as possible from study to study to allow for qualitative comparisons of data between studies. This approach has allowed us to make two useful observations.

### The disease course is remarkably consistent

Data in table 2 show the average age at onset and end-point for the vehicle control group in each individual study and the average time taken for mice to show an initial 20% decline in rotarod performance. This data is shown for the whole group and is also broken down by sex. The studies are numbered according to the reference numbers in table 1. The data for onset and survival show very low within-study (intra-study) variability as shown by the low coefficient of variation (CV- standard deviation as a percentage of mean). The CV for onset ranges from 3.8% to 6.9% and for survival from 3.8% to 4.7%. For end-stage, the standard deviation is approximately 6 days compared to the average standard deviation seen for the mixed background (SJLBL6) model of 10 days [21]. The impact of this seemingly small difference on study design is large. Power analysis using GraphPad Statmate, shows that to detect a difference of approximately 10% in survival times using a Students' T test would need an n of 17 per group for an SD of 10 compared to an n of 7 for an SD of 6 days. The survival data also show excellent between study (inter-study) variability with a coefficient of variation of 1.7% across the studies listed in table 2. The inter-study variability for the onset data is higher at 5.0%, possibly reflecting variation between scorers for this subjective assessment. There are no significant differences between males and females for any of the parameters measured although female mice show a trend to reduced variability in the various readouts. This is particularly true for the rotarod data where female mice show a two-fold or more reduction in variability for time to reach a 20% reduction in rotarod performance in 4 out of 5 studies. The rotarod data are the most variable and there is the possibility of a time-related change in rotarod performance with studies 1 and 2 showing an average age at 20% decline in rotarod performance of less than 50 days and studies 4, 5 and 6 showing an average of 57.6, 58.3 and 52.0 days respectively. Whether this reflects a time-dependent change in measurement of this parameter or simply variability around the true mean is difficult to ascertain.

**Table 2:** Reproducibility between studies.

	Trial Number																	
	1			2			3			4			5			6		
	m	f	all	m	f	all	m	f	all	m	f	all	m	f	all	m	f	all
**Onset (age in days)**	74.0 (±5.2)	74.8 (±3.7)	**74.4 (±4.7)**	nd	nd	nd	77.8 (±5.1)	74.2 (±4.6)	**75.6 (±4.9)**	72.5 (±5.5)	76.3 (±2.9)	**73.5 (±5.1)**	72.4 (±4.7)	74.4 (±2.5)	**73.4 (±3.7)**	65.9 (±2.2)	68.5 (±3.5)	**66.4 (2.6)**
**CV (%)**	7.0	4.9	**6.4**				6.6	6.2	**6.5**	7.5	3.8	**6.9**	6.4	3.4	**5.0**	3.3	5.2	**3.8**
**Survival (age in days)**	nd	nd	nd	138.7 (±6.6)	134.0 (±5.4)	136.8 (±6.4)	142.0 (±7.1)	144.7 (±5.3)	**143.6 (±6.1)**	138.5 (±5.3)	142.7 (±3.9)	**139.6 (±5.3)**	136.6 (±7.2)	142.0 (±3.1)	**139.3 (±6.0)**	140.4 (±6.6)	136.5 (±0.7)	**139.6 (±6.0)**
**CV (%)**				4.3	4.7	4.7	5.0	3.7	**4.2**	3.8	2.7	**3.8**	5.3	2.2	**4.3**	4.7	0.5	**4.3**
**Rotarod (age at 20% reduction)**	48.8 (±8.7)	47.6 (±4.0)	**48.2 (±6.4)**	48.7 (±4.6)	44.6 (±2.2)	47.0 (±4.2)	nd	nd	nd	56.5 (±11.4)	60.7 (±9.1)	**57.6 (±10.5)**	59.6 (±21.2)	57.0 (±7.5)	**58.3 (±15.0)**	55.3 (±11.0)	46.7 (±3.5)	**52.0 (±10.1)**
**CV (%)**	17.9	8.4	**13.3**	9.5	4.9	9.0				20.1	15.0	**18.3**	35.5	13.2	**25.8**	19.8	7.5	**19.1**

Data shown are for the entire control group (all) for six independent efficacy studies in the SOD1^G93A^ transgenic mouse model of ALS, as well as the data for male (m) and female (f) mice in each study. Study details are given in table 1. Each parameter is measured in age (days) +/− standard deviation. ‘Onset’ is age in days at which both hind-limb splay and tremor were first obvious; ‘Survival’ is age in days to humane end-point (loss of righting reflex for more than 10 seconds or weight loss of >30% for >72 hours), ‘Rotarod’ is the age in days at which a 20% reduction in rotarod performance compared to baseline was first seen. For each parameter the coefficient of variation (CV) is also shown. This is the standard deviation expressed as a percentage of the mean. These parameters are not comparable either because different criteria were applied or the data were not collected; nd-not determined, nk-not known.

### Motor deficits are detectable very early and correlate with published patterns of synaptic remodelling at motor end plates

The data in table 2 show that SOD1^G93A^ transgenic mice exhibit a very early decline in rotarod performance, with a 20% reduction in performance from baseline occurring by day 50–60. These data are more variable than the onset and survival data - the coefficient of variation ranges from 9% to 25.8%. When the rotarod data for individual studies are plotted (figure 1) a very obvious rapid decline in rotarod performance is observed from approximately 45 days of age. These data are generated after the training period, and so do not represent acclimatisation to the apparatus. Rotarod performance generally increases in the three successive training runs (data not shown). Littermate control non-transgenic (NTG) mice do not show the same pattern of decline and indeed at the earliest timepoint shown here, they are already outperforming their SOD1^G93A^ transgenic littermates. Overlay of the rotarod data in figure 1 also highlights another interesting feature. It appears that the rate of decline abruptly slows at around 60 days of age. This rapid decline followed by a levelling off correlates with the recently described denervation of fast-fatigable fibres (days 48 to 52) followed by their re-innervation and conversion to fast non-fatigable fibres from day 60 onwards [3]. The authors of this study also described a second loss of innervation for these fast non-fatigable fibres between days 80 to 90 of age which correlates with a further decline in rotarod performance seen here (see figure 1).

### Neuromuscular Junction (NMJ) Histology

**Figure 2:**
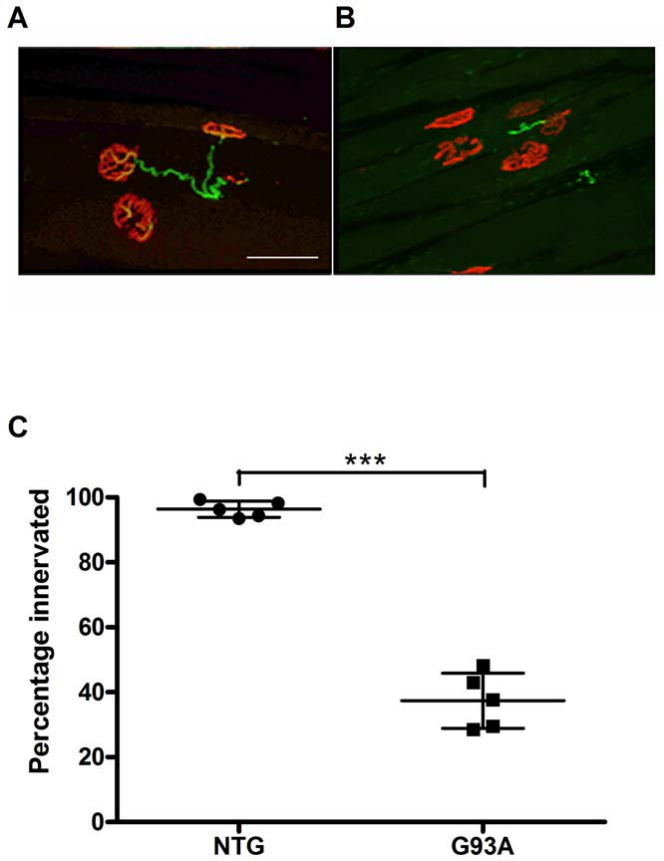
Quantification of neuromuscular junction (NMJ) innervation at 60days in SOD1^G93A^ transgenic mice. (A, B) Immunofluorescence staining of gastrocnemius muscle with α-Bungarotoxin (red) and anti-neurofilament antibodies (green). NMJ were counted as innervated when end-plates showed overlap of neurofilament and AChR staining (A, image from a non-transgenic mouse) and denervated when endplates showed absence of neurofilament labeling (B image from a SOD1G93A transgenic mouse). Scale bar 50 µM. The percentage of α-Bungarotoxin stained end-plates showing complete and partial co-localisation of neurofilament staining versus absence of colocalisation (C) is shown for 5 non-transgenic mice (NTG) and 5 SOD1G93A transgenic mice (G93A) at 60 days of age. Average +/−standard deviation is shown (NTG 96.4+/−2.5%; G93A 37.4+/−8.5%, p<0.0001, Student's T test). Graph displays result for individual animals. Median horizontal line indicates the mean value. Upper and lower horizontal lines linked by a vertical line show SD.

In order to confirm that this early decline in motor function correlated with an early loss of innervation of motor end-plates in the gastrocnemius muscle, we performed dual immunofluorescence staining of nicotinic acetycholine receptors(AChR) with α-bungarotoxin and axonal end-feet with anti-neurofilament antibodies and quantified the percentage of post-synaptic processes showing some degree of axonal co-localisation (figure 2). Overall there was an almost 60% loss of co-localised staining in SOD1^G93A^ transgenic mice at 60 days of age compared to non-transgenic mice of the same age. (NTG 96.4+/−2.5%; SOD1^G93A^ 37.4+/−8.5%, p<0.0001, Student's T test).

Given that significant motor function decline associated with an early process of NMJ loss was observed in our model, we postulated that it would be possible to detect the therapeutic effects of interventions at a much earlier stage than had previously been described, providing disease progression could be measured in a robust and quantitative manner. To this end we assessed additional measures of disease progression using quantitative gait analysis and magnetic resonance imaging of hind-limb muscle volumes.

### Gait Analysis

We embarked on a gait analysis study in a cohort of SOD1^G93A^ mice and NTG control mice using the Catwalk gait analysis system from Noldus Instruments [29]. A group of 5 SOD1^G93A^ mice and 7 age-matched NTG mice were assessed at weekly intervals from 35 days of age. The system allows for automated analysis of gait parameters following video capture and manual segmentation of step patterns. The results for 42 different gait parameters are shown in table 3 as the raw P values for the difference between SOD1^G93A^ mice and NTG mice using a Students' T test at each timepoint, for each parameter. The P value required for significance after Bonferroni correction is <0.00011 and the P values below this threshold are highlighted in bold.

**Table 3:** Raw P values (Students' T test) for 42 different gait parameters in SOD1^G93A^ mice vs NTG mice.

Age (days)	35	42	50	56	63	70	77	84	91	98	105
Intensity - fore paws	0.034	NS	0.0207	0.0083	NS	NS	NS	NS	0.0175	0.0323	NS
Intensity - hind paws	NS	NS	NS	NS	0.0202	0.0453	0.0317	0.0043	**<0.0001**	**<0.0001**	**<0.0001**
Print Width - fore paws	NS	0.0078	0.0172	NS	NS	NS	NS	0.0217	0.0088	0.0016	0.0002
Print Width - hind paws	NS	NS	NS	NS	NS	NS	NS	NS	NS	NS	0.0185
Print Length - fore paws	NS	0.0011	**<0.0001**	0.0005	NS	NS	NS	NS	0.0492	0.0244	0.0109
Print Length - hind paws	NS	0.0408	NS	NS	NS	NS	NS	NS	NS	NS	NS
Print Area - fore paws	NS	NS	0.0213	0.0014	NS	NS	NS	0.0326	0.0399	0.0034	**0.0001**
Print Area - hind paws	NS	NS	NS	0.0169	NS	0.002	0.0113	NS	0.0463	NS	NS
Stand time - forelimb	NS	NS	**<0.0001**	NS	**<0.0001**	NS	**<0.0001**	0.001	**0.0001**	0.0009	**<0.0001**
Stand time - hindlimb	NS	NS	0.0074	NS	**<0.0001**	NS	0.0016	NS	**0.0001**	0.0021	**0.0001**
Paw angle - fore paws	NS	NS	NS	NS	NS	NS	NS	0.0477	NS	NS	NS
Paw angle - hind paws	NS	NS	NS	NS	NS	NS	NS	0.0465	NS	NS	NS
Swing - forelimb	NS	NS	NS	NS	0.0314	NS	NS	NS	0.0278	0.0097	0.002
Swing - hindlimb	NS	NS	NS	NS	NS	NS	0.0061	NS	NS	0.05	NS
Stride length - front	NS	NS	NS	NS	0.039	NS	NS	0.0036	0.0045	0.0002	**<0.0001**
Stride length - back	NS	NS	NS	NS	0.0339	NS	NS	0.0005	0.0003	**<0.0001**	**<0.0001**
Duty cycle - forelimbs	NS	NS	**<0.0001**	0.0195	0.0073	NS	NS	**0.0001**	**<0.0001**	**<0.0001**	**<0.0001**
Duty cycle - hindlimbs	0.0415	NS	NS	NS	0.0181	NS	NS	0.0053	**<0.0001**	0.0007	**0.0001**
Max contact at % - fore paws	NS	NS	NS	NS	NS	NS	NS	NS	NS	NS	0.0088
Max contact at % - hind paws	NS	NS	NS	NS	NS	NS	NS	NS	NS	NS	0.0055
Swing speed - fore limb	NS	NS	NS	NS	NS	NS	NS	NS	NS	NS	NS
Swing speed - hind limb	NS	NS	0.0228	NS	NS	NS	NS	0.0193	NS	NS	NS
Stand index - fore limb	NS	NS	0.0011	NS	0.0044	NS	NS	0.0117	0.0202	0.0416	0.0193
Stand index - hind limb	NS	NS	0.0053	NS	NS	NS	NS	NS	NS	NS	0.0052
Duration	NS	NS	NS	NS	NS	NS	0.0322	NS	NS	0.0412	0.037
Step pattern #	NS	0.0173	NS	NS	NS	NS	NS	0.0248	0.0034	NS	0.0051
Step pattern Ca	0.0414	NS	NS	NS	NS	NS	NS	NS	NS	NS	NS
Step pattern Cb	NS	NS	NS	0.0372	NS	NS	NS	NS	0.0041	0.0039	0.0088
Step pattern Aa	NS	NS	NS	NS	NS	NS	0.0211	NS	0.0019	0.018	**0.0002**
Step pattern Ab	NS	NS	NS	NS	NS	NS	0.0353	NS	**0.0001**	0.0005	**<0.0001**
Regularity Index	NS	NS	NS	NS	NS	NS	NS	NS	NS	NS	0.0002
Base of support - front	NS	NS	NS	NS	NS	NS	NS	NS	NS	NS	NS
Base of support - hind	NS	NS	NS	NS	NS	NS	NS	NS	0.0156	0.003	0.0027
Print position - right	NS	0.0012	NS	NS	NS	NS	NS	NS	NS	NS	NS
Print position - left	NS	0.0214	NS	NS	NS	NS	NS	NS	NS	NS	NS
Support - zero	NS	NS	NS	NS	NS	NS	NS	NS	NS	NS	NS
Support - single	NS	NS	0.017	NS	NS	NS	NS	NS	NS	NS	NS
Support - diagonal	NS	NS	NS	NS	0.0013	NS	0.0303	0.0095	**<0.0001**	**<0.0001**	**<0.0001**
Support - lateral	NS	NS	NS	NS	NS	NS	NS	NS	NS	NS	NS
Support - girdle	NS	NS	NS	NS	NS	NS	NS	NS	NS	NS	NS
Support – 3 limbs	NS	NS	NS	NS	0.0024	NS	0.0353	NS	0.0037	0.0003	**<0.0001**
Support – 4 limbs	NS	NS	NS	NS	0.0199	NS	NS	0.0036	0.021	0.0215	0.0286

Gait parameters for SOD1^G93A^ mice and non-transgenic (NTG) mice were determined using the Catwalk system. Gait was assessed at weekly intervals. P values highlighted in bold are less than the required P value of <0.00011 following a Bonferroni correction.

**Figure 3:**
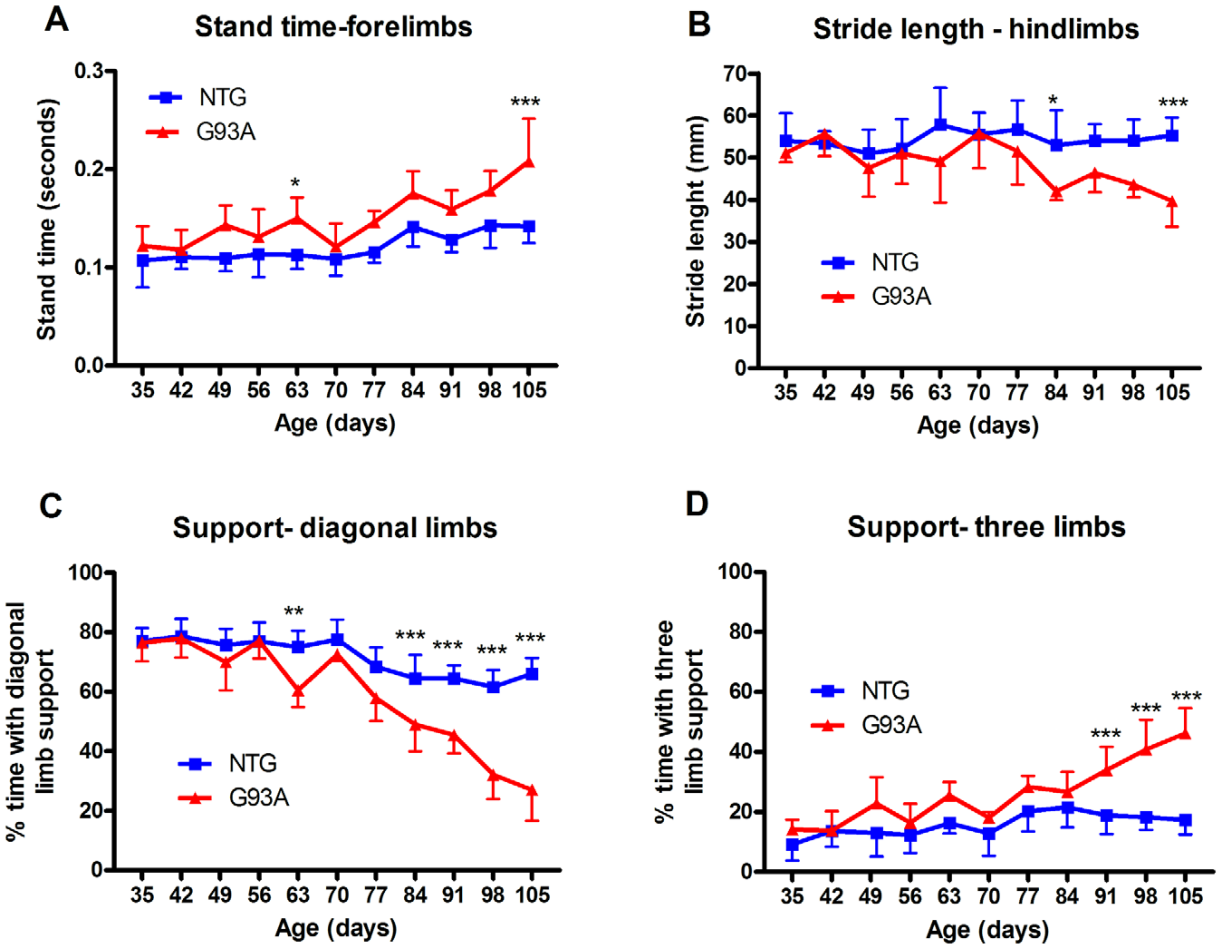
Analysis of individual gait parameters over time in SOD1^G93A^ transgenic mice. Selected gait parameters from table 3 were subjected to two-way ANOVA with Bonferroni post-tests. Average stand time (a) in seconds, hindlimb stride length (b) in mm, use of diagonal support (% of time) and use of three limbs for support (% of time) +/− standard deviation for SOD1^G93A^ mice versus non-transgenic (NTG) control mice at weekly intervals between 35 and 105 days of age are shown. Asterisks indicate p<0.05 (*), p<0.01 (**) and p<0.001 (***) using bonferroni post test following two-way ANOVA on raw data (a, b) or logit transformed data (c, d). The majority of differences were detectable post-onset of classical signs (∼75 days).

This analysis indicated that there were some parameters showing small but significant changes at early time-points, although the majority of changes appeared after the onset of visible clinical signs (∼74 days) indicating that gait analysis is not as sensitive as the rotarod test for detection of early motor deficits in this model. Some of the altered gait parameters are shown in figure 3. Stand time is the duration of the stance phase for a particular limb in seconds and this is marginally increased at 63 and 105 days in SOD1^G93A^ mice (63days; 0.01±seconds in NTG vs 0.15±0.02 mm in SOD1^G93A^, p<0.05, 105 days; 0.14±0.02 seconds in NTG vs 0.15±0.02 mm in SOD1^G93A^, p<0.001, two-way ANOVA with Bonferroni post-test). The most commonly assessed gait parameter in mouse models of ALS is stride-length and significant differences in stride length of hind-limbs were seen at 84 and 105 days of age (figure 3b 84 days; 53.0±8.3 mm in NTG vs 42.0±2.1 mm in SOD1^G93A^, p<0.05, 105 days; 55.3±4.2 mm in NTG vs 39.7±6.1 mm in SOD1^G93A^, p<0.001, two-way ANOVA with Bonferroni post-test). Gait patterns were also disturbed, with SOD1^G93A^ mice progressively spending less time supporting themselves with diagonal limbs (figure 3c) with a concomitant increase in the use of three limbs to support their weight (figure 3d), which may reflect a reduced stability of gait. It is interesting to note that some of the gait parameters show initial changes only to recover later on, whilst some of this may be attributable to noise, it may also be the case that dennervation followed b reinnervation [3] is responsible for a loss and then recovery reflected in the changing significance levels.

### MRI Imaging of hind-limb muscle volume

**Figure 4:**
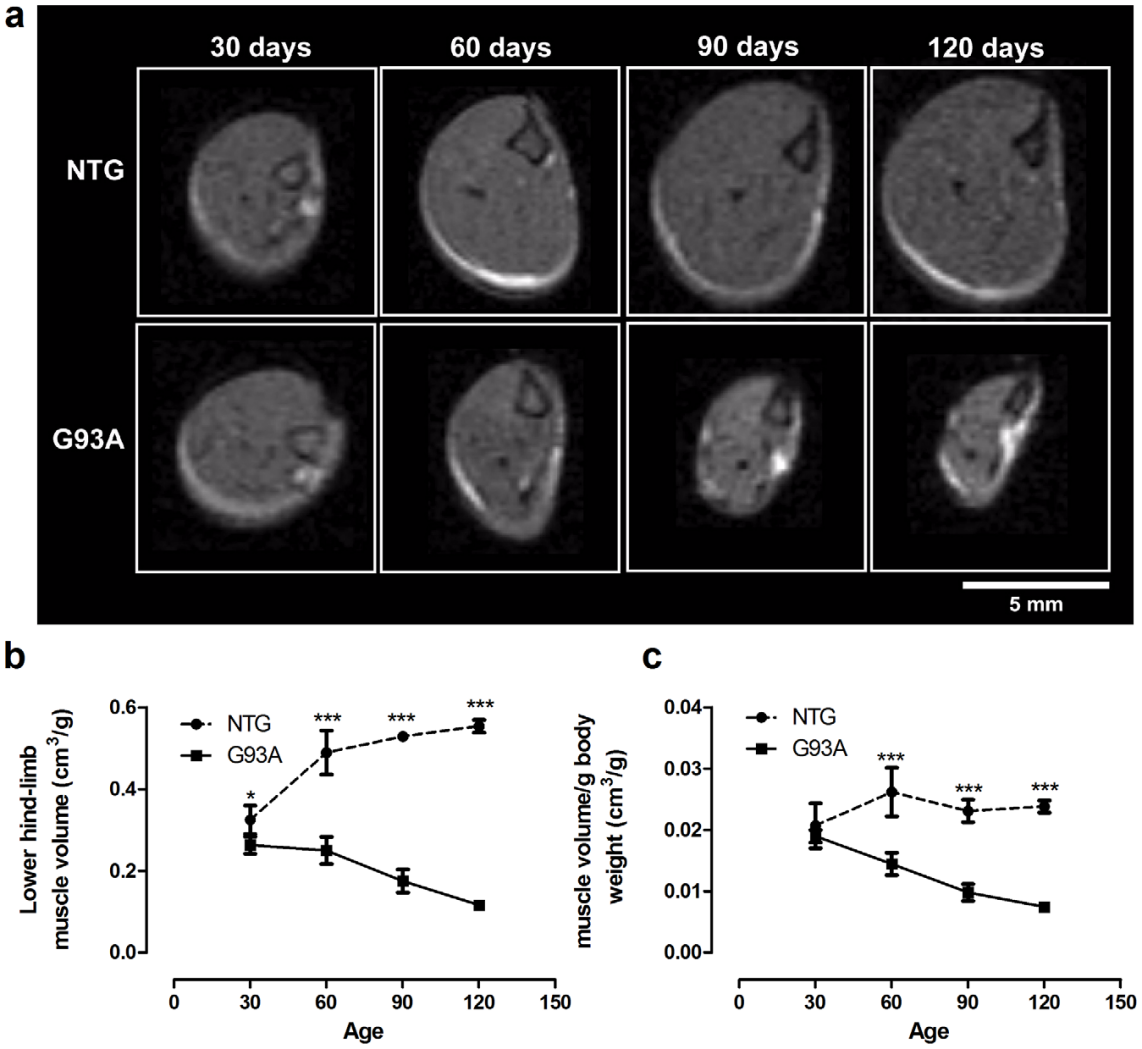
MRI detects muscle atrophy in SOD1^G93A^ transgenic mice. T2 weighted structural scans of hindlimb at 30, 60, 90, 120 days of age (a) were used to calculate lower limb volume (knee to ankle), n = 5 per group (b), which was significantly reduced in SOD1^G93A^ transgenic mice compared to non-transgenic (NTG) littermates at all timepoints (p<0.05 at 30 days, p<0.001 at 60, 90, 120 days, two way ANOVA with Bonferroni post test). The change in lower limb muscle volume as a function of weight (c) is also shown, indicating the initial difference in muscle volume observed at day 30 may be due to the smaller size of the SOD1^G93A^ transgenic mice.

An MRI study was conducted to assess lower hind-limb muscle volumes in five SOD1^G93A^ transgenic mice and five litter matched NTG mice at 30, 60, 90 and 120 days of age. Structural (T2 weighted) MRI scans of hindlimbs were used to determine lower limb volume (figure 4). Significant differences between SOD1^G93A^ transgenic mice and NTG littermates were observed at all timepoints. At 30 days these may reflect overall differences in size between NTG and SOD1^G93A^ mice but the changes thereafter appear to be due to muscle atrophy. In the SOD1^G93A^ mice, average lower limb volume reduced by 5% between 30 and 60 days, 33% between 30 and 90 days and by 56% between 30 and 120 days. This contrasts with an increase in muscle volume of 53% between days 30 and 60, 33% between days 30 and 90 and 56% between days 30 and 120 in NTG mice.

## Discussion

We have shown that the SOD1^G93A^ mouse model of ALS on a defined background is highly predictable and shows low variability in a number of readouts, particularly survival. In addition there are early and significant changes in motor performance which can be reliably detected. We believe this has important implications for the testing of therapeutic strategies and for basic investigations of disease biology in this model. Firstly, use of mice with an undefined genetic background is well recognised as a potential confounding issue in preclinical drug screening [30]. Indeed the use of the hybrid SJLxC57 SOD1^G93A^ transgenic mice has identified a variety of potentially confounding factors which can contribute to background noise and lead to false positive effects, if not adequately controlled, as well as requiring studies with large n numbers of 24 mice per group [21]. The results we show here for a significantly reduced variability in time to reach end-stage correlate with those obtained previously for a defined C57BL/6 background with this transgene [31] where the standard deviation of the end-point on the C57BL/6 background was 7.5 days compared to 6 days in our model and 11.2 days in the SJLBL6 background [21]. This reduction in variability has a significant impact on the number of animals required to conduct pharmacological studies. It is possible that the variability is influenced by the precise criteria used to define the end-stage of disease and the two cited studies used slightly different criteria to define the end-stage as was used here. Scott et al used “inability of a mouse to right itself in 30 s after being place on its side”, Heiman-Patterson et al used “paralysis of one hind limb, or were unable to right themselves in 30 s when placed on their side” whereas we used “inability to right within 10 seconds of being placed on their back or weight loss of >30% for 72 hours”. In practice the time cut-off for measuring loss off righting reflex (10 s or 30 s) would not be expected to make a substantial difference in when the end-stage of disease is recorded, as discussed in the recent ‘Guidelines on Preclinical models of ALS’ [23]. In addition the weight loss cut-off used in our criteria was implemented in less than 10% of mice and again would not be expected to substantially influence the variability in reaching end-stage. It is possible that a proportion of the variability may be attributed to these different criteria however when Heiman-Patterson et al applied the same criteria to both C57BL/6 and SJLBL6 background SOD1^G93A^ transgenics there was still a substantial reduction in the SD of time to reach end-stage [31].

We also show that the between-study variability for this readout is very low. With a variety of different dosing regimes (table 1), the inter-study variability for time to reach end-stage is just 1.7% (CV). This greatly facilitates the identification of real positive effects in our model. In addition, the potentially confounding differences in survival seen between male and female mice on the mixed background are not seen in our C57BL/6 line, again confirmed in a previous study [31]. The only consistent difference between male and female mice in our studies is that female mice show substantially reduced variability in time to reach a 20% reduction in rotarod performance in 4/5 studies (table 2). This is the opposite of the trend observed in the SJLBL6 line [32] which again may be explained by strain differences. The reason for this reduced variability in female C57BL/6 mice is unknown but gender differences in voluntary wheel-running have been previously described in C57BL/6 mice [33]. In addition the rotarod data showed the greatest variability overall both within studies and between studies.

The second major impact of our findings is that the early defects in rotarod performance described here bring into question when ‘onset’ actually occurs in this model. Typically a figure of 90 days is quoted for onset in the C57×SJL background as the point at which visible signs such as tremor and hind-limb splay defects are detectable. We observe these signs at about 75 days of age in the C57BL/6 background. Defining onset as the point at which these crude measures of tremor and hind-limb splay become obvious is inappropriate in our view. These mice have a very high level of mutant SOD1 transgene expression and the mice reach end-stage at about 140 days of age, whereas normal C57BL/6 mice can enjoy an average lifespan of over 800 days [34]. This suggests the disease process is aggressive. A review of relevant published work indicates that many behavioural, electrophysiological and neuropathological changes are occurring in mutant SOD transgenic mice from the very earliest stages of development (Table 4).

**Table 4:** Summary of studies describing ‘pre-symptomatic’ changes in SOD^G93A^ mice.

Process	Age	Strain	Reference
*Behavioural/Motor function*			
Delay in development of righting and hind-paw grasping reflexes	P1–7	SOD1^G85R^ C57BL/6J	[39]
Delay in development of locomotor abilities	P1–7	SOD1^G93A^ C57×SJL	[40]
Reduced open-field motor function	P45	SOD1^G93A^ C57BL/6J	[36]
Reduction in muscle strength and co-ordination	P56	SOD1^G93A^ C57×SJL	[35]
Altered gait (treadmill)	P56	SOD1^G93A^ C57BL/6J	[37]
*Electrophysiology*			
Rhythmic motor activity difficult to induce at lumbar levels ex vivo	P1–7	SOD1^G85R^ C57BL/6J	[39]
Motor neurones have lower input resistance and higher membrane capacitance	P6–10	SOD1^G85R^ C57BL/6J	[41]
Hypoglossal motor neurones show increased excitability and synaptic remodelling. Similar changes in interneurons of the superior colliculus	P4–10	SOD1^G93A^ C57×SJL	[40]
Decrease in amplitude and conduction velocity, increase in latency in sciatic nerve	P20–40	SOD1^G93A^ C57×SJL	[51]
Loss of functional motor units by electromyography	P47	SOD1^G93A^ C57×SJL	[38]
*Neuropathological*			
Vacuolation and mitochondrial swelling in anterior horn neurones of lumbar spinal cord	P14	SOD1^G93A^ C57BL/6J	[42]
Increased oxidative stress (protein carbonyl content) in spinal cord	P30	SOD1^G93A^ C57×SJL	[7]
Fragmentation of Golgi apparatus	P31	SOD1^G93A^ C57×SJL	[43]
Significant denervation of motor end-plates in hind-limb muscles	P47–50	SOD1^G93A^ C57×SJL	[2], [3], [52]
Astrocyte activation (increased glial fibrillary acidic protein (GFAP) staining) in spinal cord	P47	SOD1^G93A^ C57×SJL	[2]
Microglial activation in spinal cord	P80	Not stated	[53]

Studies showing ‘pre-symptomatic’ defects or changes in motor function, electrophysiology and neuropathology in mutant SOD transgenic mice are detailed. In all cases the earliest age at which mice show the changes is quoted as the postnatal (P) age in days.

Our data show a robust and reproducible, decline in motor function as measured by rotarod performance from approximately 40 days of age, over a month prior to the onset of visible clinical signs of disease, although this parameter is the most variable of those measured in this study. A reduction in motor performance has been defined by other groups at similar ages [34], [35], [36], [37], which correlates with a loss of functional motor units as measured by electromyography [38] and the onset of denervation of motor end-plates [2], [3], [17]. At 60 days of age in our model, the hind-limb gastrocnemius muscle shows a 60% loss in the number of post-synaptic sites showing innervation, compared to wild-type mice. This is a full two weeks prior to the onset of visible signs of tremor and hind-limb splay defects. These changes correlate with a substantial reduction in lower limb volume in SOD1^G93A^ transgenic mice, with a reduction between postnatal days 30 and 60 of 5%, whereas non-transgenic littermates show an increase of 53% in lower hind-limb volume over the same time.

Abnormalities in motor function have been detected even earlier. A delay in the development of motor reflexes has been described in both SOD1^G85R^ C57BL/6J and in SOD1^G93A^ C57×SJL mice between postnatal (P) days 1–7 [39], [40] suggesting that the development of the motor system itself is impaired. These reflexes eventually develop to the level seen in control mice by around P10. At similar time-points, a variety of studies have described electrophysiological changes occurring in spinal cord and motor cortex of mutant SOD transgenic mice [39], [40], [41]. It is attractive to hypothesize, as suggested by Amendola et al. [39] that the motor system compensates for an early defect in development by some unknown adaptation, but that this adaptation eventually fails leading to motor neuron degeneration, starting with a dying back axonopathy.

Early pathological changes that could give some clues to early disease mechanisms are the presence of vacuoles in dendrites and mitochondria of motor neurones which occur as early as P14 [42]. Of the other major pathways implicated in mutant SOD toxicity, oxidative stress occurs early, around P30 [7] with fragmentation of the Golgi apparatus at P31 [43] and early signs of inflammation such as astrocytic activation occurs around the same time as the initiation of denervation at P47 [2]. Motor neurones isolated from embryos of SOD1^G93A^ transgenic mice display impaired fast axonal transport, highlighting this as one of the earliest pathological events so far described [15].

Interestingly, quantitative measurement of multiple gait parameters did not yield any readouts with a substantial early change in function at similar time-points to the observed reduction in rotarod performance. The most robust changes were most obvious at later stages of the disease process after the onset of visible clinical signs. The rotarod test assesses a variety of motor and non-motor functions simultaneously. Co-ordination, balance, strength, stamina and determination are all probed. Given the preferential loss of processes innervating fast-fatigable fibres [3] and the substantial loss of muscle mass, we conclude that muscle strength is the major determinant of the decline in rotarod performance.

Other groups have described standardised methods for assessing a variety of motor function readouts in the hybrid SJLBL6 SOD1^G93A^ transgenic mouse [32], [44] and have concluded that rotarod and footprint analysis were the most informative methods [32] or have identified the optimal timepoint at which to apply specific tests [44].However they do not offer an alternative early screening paradigm as described here.

**Figure 5:**
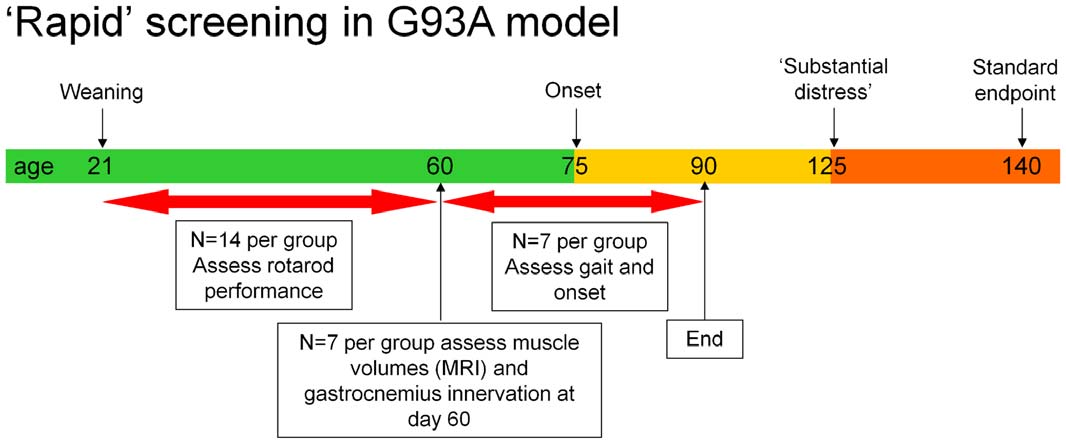
A short term protocol for preclinical pharmacology studies in SOD1^G93A^ transgenic mice. Effects on rotarod performance are assessed between days 35 to 60, analysis of hind-limb muscle volume and immunohistochemistry to assess the degree of gastrocnemius innervation at day 60 in half of the cohort and the remaining cohort being assessed for motor function using the catwalk gait analysis system and for time to onset. The n numbers have been selected on the basis of the data presented in this paper. The most variable readout requiring the largest n is the time to reach a 20% reduction in rotarod performance with a coefficient of variation (CV) ranging from 9.0% to 26.6% and an average CV of 17%. Fourteen mice per group are required to detect a shift of 10 days in this initial decline in rotarod performance with a power of 80% (two-tailed Student's T test, α = 0.05, β = 0.8). Power analysis shows an n of 7 is sufficient to detect a 20% change in muscle volume assessed using MRI based on the 60 day data for SOD1^G93A^ mice shown in figure 4, where hind-limb muscle volume was 0.25 cm^3^+/−0.033 (SD) on day 60 (Student's independent T test, two-tailed, α = 0.05 and β = 0.8). From figure 3 the percentage of innervated neuromuscular junctions in the gastrocnemius muscle of 60 day old SOD1^G93A^ transgenic mice was 37.4+/−8.5% (SD). Assuming a 15% increase in this level of innervation to 52.4%, an n of 7 per group would give 80% power (β = 0.8) at a significance level of 0.05 (Students T test, two-tailed). For onset, 7 mice per group is sufficient to detect a difference of 1 week in onset of clinical signs of disease (two-tailed Student's T test, α = 0.05, β = 0.8). All power analyses were performed with G*Power version 3.0.3.

Taken together, the data demonstrate that the disease process is active from the earliest ages and standard notions of disease onset should be reconsidered. In view of this, we advocate a screening protocol based on assessing therapeutics in this early window of loss of motor function and hind-limb muscle innervation, with a view to reducing the number of animal experiments which have to be taken through to phases of disease which cause the most distress. This approach has an added advantage of allowing a more rapid assessment of therapeutic utility and requiring fewer resources in terms of manpower and costly therapeutic reagents. The following protocol, summarised in figure 5 with power calculations, is recommended.

1/ Use 14 litter and sex matched mice in each of two groups (control dosed and active dosed), dosed from day 21 onwards (weaning), with weighing at each dose.

2/ Assess from day 35 twice per week for motor performance using rotarod testing following 3 days consecutive training on ∼days 30–33.

3/ At day 60 assess hind-limb muscle volumes using MRI in 7 mice per group and collect tissue from the same mice to assess percentage innervation of motor end-plates in the gastrocnemius muscle.

4/ Assess the remaining 7 mice per group, 3 times per week for onset of clinical signs from 60 days of age to day 90 (if necessary) and gait analysis using the catwalk gait analysis system at 63, 77 and 91 days of age (9, 11, and 13 weeks). Collect tissue at day 91 for further analysis of histological readouts.

This protocol not only allows for reduction of animal numbers and refinement of the experimental procedure with respect to animal welfare in line with the principles of the 3Rs, but allows for assessment of multiple quantitative measures of disease progression relating to defined neuropathological and physiological changes occurring in muscle which relate to the underlying pathophysiology of the disease.

The main criticism of this approach is that mechanisms active early in the disease may not be responsible for later progression. The model we propose allows clearer evaluations of therapeutic interventions on the onset of muscle denervation. It can be supplemented by additional studies to determine whether the time taken to reach end-stage is affected and also if treatment initiated later in the disease course has a differential effect, but these studies can be rationally restricted to those therapies which display some therapeutic benefit in the early stages of disease. We see this screening paradigm as a tool for accelerating therapeutic assessment in mouse models of ALS using robust and quantitative methodology with the intention of bringing new therapies to the clinical arena more rapidly.

## Materials and Methods

### Ethics Statement

All mouse experiments were carried out in accord with the Animals (Scientific Procedures) Act 1986 under a UK Home Office project license number 40/3089, reviewed and approved by the Sheffield University Ethical Review Committee Project Applications and Amendments Sub-Committee of the Sheffield University Ethical Review Committee and by the Animal Procedures Committee (London, UK). Animals were housed and cared for according to the Home Office Code of Practice for the Housing and Care of Animals Used in Scientific Procedures. The ARRIVE guidelines have been followed in reporting this study [45].

### Transgenic C57BL/6 SOD1^G93A^ model of ALS

Mice were originally obtained from the Jackson Laboratory, B6SJL-Tg (SOD1-G93A)1Gur/J (stock number 002726) and were subsequently backcrossed onto the C57BL/6 background (Harlan UK, C57BL/6 J OlaHsd) for >20 generations to create a line on an inbred genetic background. As well as reducing variability, this avoids any complications arising from the presence of a splice-site mutation in the *Dysferlin* gene found in the SJL background which leads to the development of a spontaneous myopathy [46]. The SOD1^G93A^ transgene is maintained as a hemizygous trait by breeding hemizygous males with wild-type females (C57BL/6J OlaHsd, Harlan UK). Hemizygous males for breeding are available from our facility upon request.

### Genotyping and Copy Number Analysis

Mice were identified by ear clipping and the ear tissue was retained for extraction of genomic DNA using the DNeasy Tissue Kit (Qiagen). Genotyping PCRs were performed on genomic DNA in a 25 µl volume with ABgene ReddyMix (ABgene, Epsom, UK), 150 nmol each of human SOD1 primers (forward 5′-CATCAGCCCTAATCCATCTGA-3′, reverse 5′-CGCGACTAACAATCAAAGTGA-3′) and control interleukin-2 receptor (IL-2R) primers (forward 5′-CTAGGCCACAGAATTGAAAGATCT-3′, reverse 5′-GTAGGTGGAAATTCTAGCATCATC-3′). Following PCR and agarose gel electrophoresis (3% agarose gel), IL-2R PCR products were visualised at 324 bp and human SOD1, if present at 236 bp.

Mice positive for transgene expression were then subjected to copy number analysis by quantitative PCR (Q-PCR) using 12.5 ng cDNA, 1× SYBR Green PCR Master Mix (Applied Biosystems, Warrington, UK), 2 pmol of human SOD1 primers (forward 5′-CCAAGGAGCAGATCATAGGGC-3; reverse 5′-AGAGCATTGGAGAAGGCAGG-3′) in a total volume of 20 µl. Following an initial denaturation at 95°C for 10 min, DNA was amplified by 40 cycles of 95°C for 15 sec and 60°C for 1 min, on an MX3000P Real-Time PCR System (Stratagene). ddCt values were compared to a reference DNA sample containing only two copies of human SOD1.

### Pharmacology study design

Pharmacology studies have been run in a standard way to allow comparisons between data sets over time. In general the principles set out in ‘The design of animal experiments’ by M. Festing were followed [27]. Power analysis suggested 13–15 mice per group with two groups (vehicle and active) would detect a difference in survival ratio of 10% which is widely accepted as biologically relevant. We did not always achieve this final n number due to breeding constraints or mice dropping out of studies due to incorrect genotyping or issues unrelated to development of motor dysfunction. The breeding colony was maintained at 8–12 breeding males in pairs which increased to 15–20 breeding males in trios to generate cohorts for therapeutic studies. Cohorts of mice born 2–3 weeks apart were used in all studies. The litters were split to enable an even distribution of sex and parentage between the two comparison groups and the groups randomly assigned to treatment or control. In all cases the experimental unit was a single animal. Due to the limitations of combining male mice, the need to split animals by litter according to treatment and keeping vehicle and active dosed mice separate, mice were generally housed singly to ensure all mice had the same environment. Drugs were dosed without anesthesia as they were minor procedures where anesthesia would be more distressing than the administration itself. Mice were bred in a Specified Pathogen Free facility and transferred to a conventional unit for pharmacology studies.

### Behavioural measures and rotarod test

Behavioural assessment was carried out blinded to treatment group. Rotarod training was performed over 3 days with 2 trials per day. Subsequently, the rotarod test was performed once, or more usually, twice a week at the same time of day, typically in the afternoon. On each day, mice were tested twice with a rest period in between tests. The best score was taken for analysis. The rotarod (Ugo Basile 7650) was set to accelerate from 4 to 40 rpm in 300 seconds. Latency to fall (s) was recorded in seconds for each mouse. The test was performed until mice reached a time of <5 s. For data analysis we determined the time to reach at least a 20% decline in rotarod performance, this was calculated with the post-training baseline as being 100%.

Mice were weighed with every dose or at least weekly and scored for tremor, hind-limb splay and overall neurological deficit using a scoring system similar to previous studies [47], [48], [49]. Mice were scored three times per week from day 65 until onset and then at the same time as rotarod tests. Onset was defined as the point at which defects in hind-limb splay and enhanced tremor were observed (a score of at least 1 in each category). Tremor scores were recorded individually for fore-limbs and hind-limbs when suspending the mouse by the tail. The scores were 0-normal, 1-mild tremor, 2-moderate tremor, 3- strong tremor. Hind-limb splay defects were scored at the same time with the splay score recorded independently for left and right hind-limbs on a scale of 0–4 with 0 representing normal, 1 representing mild defect, 2 moderate, 3 strong and 4 paralysis of hind limb.

From 125 days of age onwards mice were closely monitored and scored for signs of distress daily, using a standardised distress scoring system based on appearance, provoked behaviour, weight loss and an overall neurological score. Provoked behavior is the behavior observed when the mice are first disturbed, for example by opening the cage and was scored as follows; 0-normal, bright and inquisitive mice, 1-mild impairment or exaggeration in provoked response, 2-a moderate reduction in provoked response or disinterested mice, 3-a strong reduction in provoked response and 4-a moribund animal. The neurological score was used to give a global score of the disease state of the mice for the purpose of monitoring distress levels and was scored as follows; 0-normal, 0.5-tremor and hind limb splay defect (onset), 1-abnormal gait, 2- Partial hind-limb parlysis (first signs of dragging), 3- Hind-limb paralysis plus forelimb weakness, 4- Significant fore and hind-limb paralysis.

This scoring was continued through to the humane end-point, inability to right within 10 seconds of being placed on their back or weight loss of >30% for 72 hours. Mice were provided with wetted mash in their cages from the first signs of hind limb paralysis and the eyes of mice showing signs of ocular discharge were bathed daily. At this time, or earlier if necessary, the bedding material was optimised, to reduce dust and improve ease of movement. Mice were euthanased with an overdose of anaesthetic (intraperitoneal injection of approximately 20 ml/kg pentobarbitone).

### Immunofluorescence staining

Mice were euthanized by dislocation of the neck (schedule 1 method following the Code of Practice for the Humane Killing of Animals under Schedule 1 to the Animals (Scientific Procedures) Act 1986) and both gastrocnemius muscles were immediately dissected out. Whole muscles were post-fixed in 4% paraformaldehyde for 20 min, followed by cryopreservation through 3 successive 5 minute incubations in 5, 10 and 15% sucrose. Specimens were incubated overnight in 20% sucrose then embedded in OCT embedding matrix. Thirty five-micrometer-thick longitudinal sections were collected on Superfrost Plus Slides (CML). Tissue sections were dried, permeabilised in blocking solution (0.5% triton X-100, SIGMA-Aldrich, 5% BSA in PBS) at 37°C for 2 hours. Rabbit polyclonal anti neurofilament 145 (Chemicon) was diluted (1∶1000) in the same blocking solution and incubated at 4°C, overnight. Anti rabbit alexa 488 conjugated secondary antibody was applied on the sections together with α-bungarotoxin-tetramethylrhodamine conjugate both at a 1∶1000 dilution in PBS; 1%BSA and incubated for 2 hours at room temperature before washing and mounting using Mowiol mounting medium. Sections were examined under an Axio microscope (Zeiss) to visualise stained end-plates. Innervated (yellow) or denervated (red) end-plates were counted on apoptome 35 µm Z-stacks as described by [2].

### Catwalk gait analysis system

The catwalk gait analysis system version 7.1 was used to capture gait parameters in groups of 5 SOD1^G93A^ transgenic mice and 7 non-transgenic mice. Mice were tested weekly from 5 weeks of age to 15 weeks of age. They were placed on the catwalk apparatus in complete darkness and the recording of gait patterns performed in a separate room. Multiple runs were recorded for each mouse and three selected for analysis. Processing of gait data was performed using the dedicated software with the assignment of limbs performed manually and subsequent automated calculation of gait parameters.

Stand time is the duration of the stance phase for a particular limb in seconds, stride length is the distance between consecutive foot placings in mm and support on diagonal limbs or three limbs is the percentage of time the mouse spends supporting its weight on diagonally opposite limbs or on a total of three limbs.

### Magnetic resonance imaging

Five female transgenic C57BL/6 SOD1^G93A^ and five age matched female non-transgenic littermates were blind imaged in a 7 Tesla magnet (Bruker BioSpec^AVANCE^, 310 mm bore, MRI system B/C 70/30), with pre-installed 12 channel RT-shim system (B-S30) and fitted with an actively shielded, 116 mm inner diameter, water cooled, 3 coil gradient system (Bruker BioSpin MRI GmbH B-GA12. 400 mT/m maximum strength per axis with 80 µs ramps) to assess lower hind limb [4] muscle volumes at postnatal (P) day 30, 60, 90 and 120.

Animals were placed in a custom built Perspex magnet capsule and imaged under gaseous anaesthesia (1–1.5%, flow rate 0.8–1.0 L/min continuous inhalation through a nose cone). Anaesthetic level was controlled on the basis of respiratory parameters; monitored using a pressure sensitive pad placed under the subject's chest (SAII Model 1025 monitoring and gating system). Inside the capsule, a non-magnetic ceramic heated hot air system (SAII - MR-compatible Heater System for Small Animals) and rectal probe, integrated into the physiological monitoring system maintained the temperature of the animal. All animals were euthanized at the 120 day time point.

A ^1^H birdcage volume resonator (Bruker, 300 MHz, 1 kW max, outer diameter 114 mm/inner diameter 72 mm), placed at the iso-centre of the magnet was used for both RF transmission and reception. A workstation configured for use with ParaVision™ 4.0 software operated the spectrometer. Following field shimming, off-resonance correction and RF gain setting a tri-plane FLASH sequence (TR = 100 ms, TE = 6 ms, Flip angle  = 30°, Av = 1, FOV = 40 mm*40 mm, Slice thk = 2 mm, Matrix = 128*128) was used for subject localisation. From this a fast (∼5 min) 3D FISP sequence (TR = 8/1200 ms, TE = 4 ms, FOV = 40 mm*40 mm*40 mm, Matrix = 256*256*128) allowed low SNR visualisation of the hind limb area and thus planning of 21 axial high SNR single Spin Echo images (TR = 3200 ms, TE = 7.5 ms, Av = 1, FOV = 40 mm*40 mm, Slice thk = 1 mm, Matrix = 256*256) covering the entire lower hind limb and pelvic region. No fat suppression was used to maximise muscle/fat contrast difference for easy segmentation.

For data processing, a macro built into ParaVision 4.0 was used for manual segmentation of muscle from fat and bone across all slices in 3 regions (the left and right hind limb and the pelvic region). Using the scan FOV setting and the slice thickness allowed for volume conversion of segmented data.

### Statistics

GraphPad Prism version 3.04 was used for all statistical analyses (GraphPad, San Diego, CA, USA). Time to onset was analysed by Student's T test, time to loss of righting reflex by Survival analysis (logrank method), rotarod and weight data were analysed using two-way ANOVA. For power analysis GraphPad Statmate or G*power3 [50] was used. All data are represented as mean and standard deviation unless otherwise stated.
